# Can Intensity-Modulated-Radiotherapy Reduce Toxicity in Head and Neck Squamous Cell Carcinoma?

**DOI:** 10.3390/cancers9100135

**Published:** 2017-10-06

**Authors:** Julie van der Veen, Sandra Nuyts

**Affiliations:** Department of Oncology, Radiation-Oncology, KU Leuven, University of Leuven, University Hospitals Leuven, 3000 Leuven, Belgium; julie.vanderveen@uzleuven.be

**Keywords:** IMRT, 3DCRT, head and neck cancer, HNSCC, toxicity, xerostomia, radiotherapy

## Abstract

Intensity modulated radiotherapy (IMRT) is a modern radiotherapy technique that was implemented in the mid-1990s. It allows closer shaping of dose, to target volumes, thereby sparing organs at risk (OARs). Before the IMRT-era, two-dimensional radiotherapy (2DRT) and later three-dimensional conformal radiotherapy (3DCRT) were the techniques of choice, but this robust way of irradiating caused more normal tissue to receive a higher dose. Radiation of cancers in the head and neck region is complex because of close proximity to critical normal tissue and the large target volumes that need to be treated at high doses. IMRT offers an elegant solution compared with 3DCRT and surgery because it allows organ preservation and improved function preservation. In this manuscript, we review the rationales for IMRT, with an emphasis on toxicity outcomes compared with 3DCRT. We performed a review of the literature and looked at the most important randomised controlled trials comparing IMRT with 3DCRT. We conclude that IMRT is safe in regard to disease outcome, and that it allows better sparing of normal tissue, thereby causing less toxicity, resulting in a smaller impact on quality of life compared with conventional radiotherapy in the treatment of head and neck cancer.

## 1. Head and Neck Cancer

Head and neck cancer (HNC) is the seventh most common cancer worldwide with 550,000 new cases every year. It is, furthermore, the seventh most common cause of death, resulting in 380,000 deaths annually [1]. Risk factors for the development of cancers occurring in the head and neck region are a history of smoking and alcohol exposure. In developed countries where a decrease is seen in smoking and alcohol exposure, there is a decrease in HNC incidence in general. However, the incidence of oropharyngeal cancer (OPC) at first stagnated, and is now, even increasing due to a different aetiology, namely infection with Human papilloma virus type 16 (HPV-16) [2].

HNC is usually diagnosed in a locally advanced but curable stage. This frequently requires a multimodal treatment approach comprising surgery, followed by radio(chemo)therapy or radio(chemo)therapy alone as definitive treatment. The treatment of choice depends on multiple factors such as tumour grade, stage and localisation, nodal involvement, patient characteristics and impact of the treatment [3]. Radio(chemo)therapy holds the potential for better functional outcomes compared to surgery, especially in locally advanced cancers where surgery could be mutilating. Treatment intensification with the addition of chemotherapy or by intensification of radiotherapy, has improved survival [4,5] but also toxicity.

## 2. Advances in Radiotherapy Techniques

### 2.1. Conventional Radiotherapy Techniques

At first, robust, simple-shaped radiation fields based on bony anatomy were aimed at the tumour to be sure the tumour was irradiated sufficiently. This was the so-called two-dimensional radiotherapy (2DRT). Large volumes of normal tissue were irradiated using this technique, causing important toxicity such as xerostomia, dysphagia and fibrosis of the skin. Since then, radiotherapy techniques have improved significantly to fit the radiation beams closer around the target volume, thereby lowering the dose to the surrounding organs at risk (OARs). These technological advances gained momentum when computed tomography (CT) scans were introduced in the late 1970s. The possibility to see the tumour and OARs more clearly also changed the way in which radiotherapy (RT) was delivered. In the 1980s, three-dimensional conformal radiotherapy (3DCRT) was implemented. Using CT scan information, the tumour and OARs could be seen, especially in the head and neck region, this has many advantages because of its complex anatomy. Furthermore, there are often multiple target volumes with complex shapes, including the primary tumour, pathological lymph nodes and elective nodal regions which are in close relation to vital structures such as the spinal cord and brainstem. Other OARs such as the parotid and submandibular glands, oral mucosa, thyroid gland and swallowing structures are also in close proximity to the target volume receiving a high dose. With 3DCRT the radiation beams are formed to fit the size and shape of the tumour better, using a multileaf collimator (MLC). This allows the radiation beam to fit the shape of the tumour, sparing surrounding normal tissue better than 2DRT. 3DCRT still causes significant volumes of normal tissue to receive a high radiation dose because RT is delivered in approximately three fields with a uniform dose in each field.

### 2.2. IMRT

To compensate for the imperfections of 3DCRT, dynamic MLCs were designed so the beams could have a different shape when coming from different angles. The intensity of the beam could also be modified, giving this new technique its name; intensity modulated radiotherapy (IMRT). It was implemented in the 1990s and has become a widespread technique since then. This more precise technique allows not only sparing of OARs, but also makes it possible to deliver inhomogeneous doses which allows simultaneous boosting of the tumour, and could facilitate dose escalation in certain regions of the tumour in the future. Volumetric modulated arc therapy (VMAT) is a specific type of IMRT [6]. It also uses dynamic MLCs, but using this technique the MLCs move while the head of the RT machine moves around the patient to allow continuous adjustments, ensuring the target volume is always accurately irradiated. Another advantage compared to IMRT is that this technique is faster.

## 3. Disease Outcome

There is no doubt that the most important outcome for cancer patients is overall survival. Using more conformal radiotherapy techniques to reduce toxicity implies treating smaller volumes. Target volume definition becomes more crucial since smaller margins are used. In theory, regions which are at risk for harbouring tumour cells could be missed if margins are set too tight, causing a so-called geographical miss. This stresses the importance of target volume delineation. For this review, we only included randomised controlled trials because they offer the best evidence.

Nutting et al. [7] published the results of their phase 3 multicentre randomised controlled trial in 2011. They included 97 patients with pharyngeal squamous-cell carcinoma, randomised to receive either conventional radiotherapy with parallel opposed lateral fields or parotid sparing IMRT. They verified at the 24-month follow-up, that there were no significant differences in loco-regional control (LRC) or overall survival (OS) between the two groups.

Gupta et al. [8] performed a similar trial with 60 patients, of which 28 were treated with 3DCRT and 32 with IMRT. They published their results in 2012. At a median follow-up of 40 months, the 3-year Kaplan-Meier estimates for LRC were 88.2% and 80.5% (*p* = 0.45) for 3DCRT and IMRT respectively. OS rates were 70.6% and 68% (*p* = 0.81) respectively. These differences were also not significant.

Ghosh-Laskar et al. [9] performed a comparable trial with 59 HNC patients and published their results in 2016. With a median follow-up of 70 months, they saw no significant difference in LRC and OS between patients treated with 3DCRT or IMRT. The 5-year LRC rates for 3DCRT and IMRT were 62.9% and 69.2% respectively (*p* = 0.2). Five year OS was 50.7% and 63.4% respectively (*p* = 1.1). The previously mentioned trials’ primary endpoints were toxicity, so although they conclude that IMRT is as safe as 3DCRT with regard to disease outcome, they are inadequately powered to come to this conclusion.

Peng et al. [10] looked at nasopharyngeal cancers only and compared 2DRT with IMRT. Their primary endpoints were LRC and OS. They included 616 patients with non-metastatic stage I to IVb nasopharyngeal cancer, of which 310 were randomised to receive 2DRT and 306 to receive IMRT. The 5-year local control rates differed significant in favour of IMRT, but only in the case of T4 tumours (81.5% vs. 62.2%; *p* = 0.05). Regionally, IMRT did better than 2DRT, especially in the case of N2 disease (93.9% vs. 91.4%; *p* = 0.02). IMRT also resulted in better OS than 2DRT at the 5-year follow-up (79.6% vs. 67.1%; *p* = 0.001), especially in N2 and stage III disease.

## 4. Toxicity Profile of Conventional RT vs. IMRT

### 4.1. Xerostomia

Irradiation of HNC can cause damage to the parotid and submandibular glands. This damage causes hypofunction of the salivary glands resulting in xerostomia. Xerostomia can be evaluated using questionnaires or can be quantified by measuring salivary flow either after stimulation or without stimulation. Eisbruch et al. [11] defines xerostomia as post-radiotherapy stimulated salivary flow as <25% of the pre-radiotherapy flow. IMRT has since its implementation in the 1990s been used to avoid irradiation of the parotid glands to reduce xerostomia. Several randomised controlled trials have been conducted to compare xerostomia between patients treated with conventional radiotherapy techniques, such as 2DRT and 3DCRT, and patients treated with IMRT.

Nutting et al. [7], as mentioned above, conducted a phase 3 multicentre randomised controlled trial: Parotid-sparing intensity modulated versus conventional radiotherapy in head and neck cancer (PARSPORT). Their primary objective was to assess late side-effects of radiotherapy by looking at the proportion of patients with xerostomia grade 2 or worse using the Late Effects of Normal Tissues Subjective-Objective Management Analytic (LENT SOMA) 1 year after radiotherapy. Salivary flow before and after radiotherapy was measured with and without stimulation. Using IMRT they were able to reduce the mean dose to the contralateral parotid gland significantly; 61 Gy in the 3DCRT group compared with 25.4 Gy in the IMRT group. At 12 months this resulted in significantly less patients with xerostomia grade 2 or worse in the IMRT group (15 out of 39; 38%) compared with the 3DCRT group (25 out of 34; 74%). This came down to an absolute reduction of 35%. At 24 months, this difference increased even more with an absolute reduction of 54% (83% in the 3DCRT group and 29% in the IMRT group). These results were not influenced by the tumour site, primary versus postoperative setting, disease stage or use of neoadjuvant chemotherapy. Besides these subjective parameters, salivary flow was also measured. At 12 months the unstimulated salivary flow from the contralateral parotid gland was measurable in 16 out of 34 patients (47%) in the IMRT group. Patients treated with 3DCRT had no measurable salivary flow from the contralateral parotid (0 out of 25). There was also a significant difference in the stimulated salivary flow from the contralateral parotid at 12 months. A strong relation was seen between salivary flow and grade 2 or worse xerostomia. However, there was not a perfect match, probably because patients experience xerostomia differently and because other factors can cause xerostomia, such as damage to oral mucosa and to other salivary glands due to radiation.

Pow et al. [12] compared 2DRT to IMRT in patients with nasopharyngeal cancers. The primary outcome was change in stimulated whole salivary (SWS) flow rate up to 12 months after treatment. They included patients randomly to a 2DRT-arm and an IMRT-arm. At the 1-year follow-up, 21 patients treated with 2DRT and 24 treated with IMRT were in remission and could be analysed. The mean SWS and stimulated parotid salivary (SPS) flow rates were significantly better after IMRT, than after 2DRT (*p* < 0.05). In both groups salivary flow decreased after treatment, but after IMRT SPS improved again, whereas this improvement was limited after 2DRT.

Braam et al. [13] investigated the difference in SPS flow in patients treated with conventional radiotherapy techniques (CRT) compared to IMRT in the treatment of oropharyngeal cancer. A total of 56 patients were included, of which 30 were treated with IMRT and 26 with CRT (2DRT and 3DCRT). Mean dose to the parotid glands was significantly lower in the IMRT group (33.7 Gy) compared with the CRT group (48.1 Gy) (*p* < 0.005). This resulted in a significant difference in the number of parotid flow complications at 6-weeks after treatment; 55% in the IMRT group and 87% in the CRT group (*p* = 0.002). Also at 6-months this remained significant; 56% in the IMRT group and 81% in the CRT group (*p* = 0.04).

The primary end-point of Gupta et al. [8] was the incidence of acute salivary gland toxicity grade 2 or worse as rated by a physician, based on the Radiation Therapy Oncology Group (RTOG) criteria. They concluded that the proportion of patients with grade 2 or worse acute xerostomia was significantly smaller after IMRT (19 of 32 patients, 59%) compared with 3DCRT (25 of 28 patients, 89%) (*p* = 0.009). Late xerostomia was also scored up to 36 months after treatment. At each time point there was a significantly smaller proportion of patients with xerostomia grade 2 or worse after IMRT than after 3DCRT.

Ghosh-Laskar et al. [9] also evaluated the incidence of grade 2 or worse acute xerostomia 8 weeks after parotid-sparing radiotherapy. Ipsilateral and contralateral parotids received significantly less dose in the IMRT-arm than in the 3DCRT-arm. This in turn resulted in a significantly lower proportion of patients with grade 2 or worse xerostomia after IMRT than after 3DCRT (24% vs. 53%; *p* = 0.024). Even at a follow-up of 2 and 5 years, there was a significantly smaller proportion of patients with xerostomia after IMRT than after 3DCRT.

In summary, all these studies concluded that IMRT significantly reduces the dose to the contralateral parotid gland, reduces parotid flow complications and results in less xerostomia compared to conventional techniques.

### 4.2. Mucositis

When mucosal tissue of the oral cavity is irradiated, mucositis can occur. Especially in combination with chemotherapy, the incidence increases and has an effect on oral intake, weight loss and quality of life. In a randomised controlled trial by Gupta et al. [8] the authors found no significant difference in acute mucositis between 3DCRT and IMRT in a group of 60 patients. When looking at grade 2 and 3 mucositis alone, disregarding grade 1, a difference can be seen. In this study, 22 of 28 patients (78.5%) had grade 2 mucositis after 3DCRT compared with 23 of 32 patients (71%) after IMRT. For grade 3 mucositis the difference is larger although the numbers are very small; 4 of 28 patients (14.5%) after 3DCRT compared with 2 of 32 patients (6%) after IMRT.

Vergeer et al. [14] performed a non-randomised controlled trial to compare mucositis between IMRT (91 patients) and 3DCRT, (150 patients) they found a significant difference in acute mucositis in favour of IMRT in weeks 3, 4, 5 and 12 after treatment (*p*-value ranging from 0.006 to 0.016). However, keeping in mind that this was a non-randomised, retrospective study, performed on prospectively collected data, this study of Vergeer et al. is prone to bias, and interpretation of the results should be done with care.

We also want to point out that the primary tumour site evidently has an influence on mucositis. When the primary tumour is located in or near the oral cavity such as oropharyngeal tumours, a more conformal technique such as IMRT will likely not result in less toxicity because a high dose has to be given to that region. Mucositis is determined by the high dose region, and this volume is dependent on the extent of the tumour, not the technique used.

### 4.3. Fatigue

**Figure 1:**
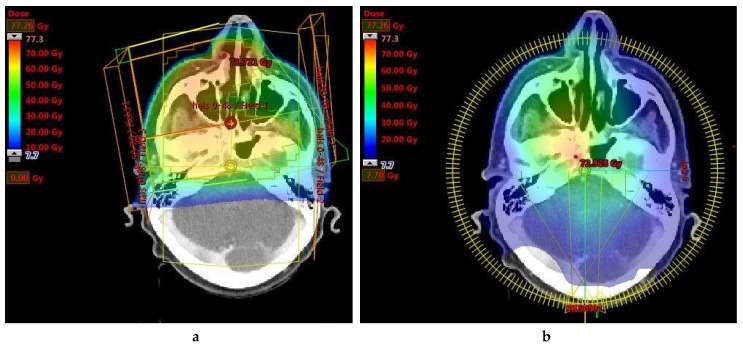
A comparison between a three-dimensional conformal radiotherapy (3DCRT) plan and a volumetric modulated arc therapy (VMAT) plan for a head and neck tumour. Notice the larger volume of the posterior fossa receiving a low dose bath in the VMAT plan. (**a**) 3DCRT; (**b**) VMAT.

**Figure 2:**
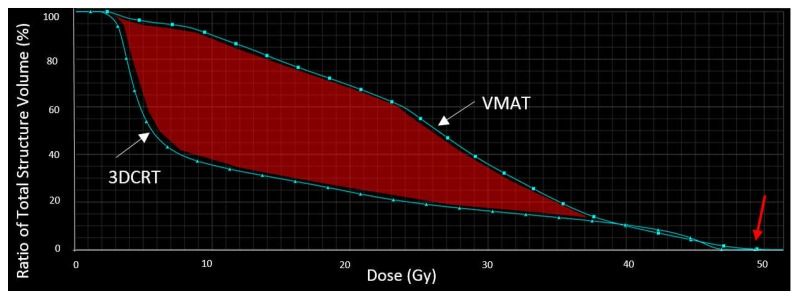
Dose-volume histogram of the dose delivered to the posterior fossa. Although the maximum dose is similar for both plans (red arrow), the average dose is higher in the VMAT plan compared to the 3DCRT plan (red surface).

Fatigue is a multifactorial symptom which could be caused by radiotherapy and chemotherapy, post-surgery, due to lower energy intake, stress or a combination of all of these. Nutting et al. [7] investigated the difference in fatigue at 12 months between patients treated with IMRT and conventional RT. They came to the unexpected conclusion that patients treated with IMRT showed significantly more signs of fatigue than patients treated with conventional RT (55 of 89; 74% vs. 18 of 44; 41%, *p* = 0.0015). They retrospectively looked at the dose to the posterior fossa and saw that it was higher in the IMRT group than in the 3DCRT group (20–30 Gy vs. 6 Gy) suggesting this could explain the higher incidence of fatigue in the IMRT group. There was no correlation with neoadjuvant chemotherapy. As an example, a comparison is shown between a 3DCRT plan and a more conformal technique; volumetric modulated arc therapy (VMAT), which is a modern type of IMRT. In Figure 1, the dose distribution is shown, illustrating that the posterior fossa receives a large low dose bath using VMAT compared to 3DCRT. In Figure 2, the dose-volume histogram (DVH) is shown, depicting that the same maximum dose is given, but that VMAT results in a higher average dose to the posterior fossa than 3DCRT, (25.5 Gy vs. 13.3 Gy respectively).

### 4.4. Dysphagia

Dysphagia is a common complaint after radiotherapy for HNC, present in up to 44% of patients treated with RT for pharyngeal cancers, 12 months after radio(chemo)therapy [15]. Patients experience this as an important side-effect, rating it with one of the highest priorities. The anatomical structures responsible for smooth and painless swallowing are, the pharyngeal constrictor muscles (PCM) and the supraglottic larynx (SGL). These two structures play an important role in the development of dysphagia after RT [16]. Nevertheless, randomised controlled trials specifically looking to spare the PCM responsible for swallowing are scarce. Nutting et al. [7] commented on dysphagia briefly, but this was not their primary outcome, as they specifically spared the parotid glands and not the PCM nor the supraglottic larynx. They concluded that at 12 months, dysphagia grade 3 or more was reported by 2 of 41 patients (5%) in the conventional radiotherapy group and 4 of 46 patients (9%) in the IMRT group. No dosimetric results are available from this trial to correlate dysphagia to dose to the PCM or SGL, although, there is evidence from smaller retrospective trials that sparing these structures can result in better functional outcomes. The question remains whether sparing of the PCM and SGL has a clinically relevant effect. Feng et al. [17] prospectively investigated this on 73 patients with oropharyngeal cancer, treated with IMRT. Patient-reported Swallowing and Eating Domain scores, observer-rated scores, and video-fluoroscopy before and periodically after radiotherapy until 2-years after treatment were used to assess swallowing. They concluded that the outcomes were only slightly worse than before therapy, which meant an improvement compared with older conventional radiotherapy techniques. However, this was not a randomised controlled trial. To provide indisputable evidence, a new randomised controlled trial has been set up that has been recruiting since 2016: A study examining whether a new radiotherapy technique (“dysphagia optimised intensity modulated radiotherapy”) will improve swallowing function after treatment in head and neck cancer patients (ISRCTN25458988) [18].

### 4.5. Weight Loss

Long-term weight loss was a secondary endpoint in the prospective randomised controlled trial of Gosh-Laskar et al. [9]. Substantial weight loss was referred to as >10% of pre-radiotherapy weight, 12 months after radiotherapy. They saw a significant difference in substantial weight loss in favour of IMRT compared with 3DCRT (5 of 24 patients; 21% vs. 11 of 22 patients; 50%; *p* = 0.038). This difference was not significant in the acute setting, although, there was a trend toward substantial weight loss during RT, also in favour of IMRT. This correlated with a higher incidence of nasogastric feeding tube requirement in patients treated with 3DCRT.

### 4.6. Hypothyroidism

Murthy et al. [19] determined the incidence of hypothyroidism after radio(chemo)therapy for locally advanced HNC and assessed this at baseline and every 3 to 6 monthly thereafter. They used the results of two randomised controlled trials in which patients were treated with 3DCRT (70Gy/35 fractions) or IMRT (66Gy/30 fractions). Of these patients, 89 were euthyroid and evaluable for post-radiotherapy hypothyroidism. Dosimetric data was available for 43 patients. A total of 55.1% (49 of 89 patients) developed hypothyroidism, reaching a peak at 1 year. There was no significant difference between the two treatment techniques regarding hypothyroidism in a general sense, although patients treated with IMRT had significantly more subclinical hypothyroidism than after 3DCRT (51.1% vs. 27.3%; *p* = 0.021). Subclinical hypothyroidism was categorised as thyroid stimulating hormone (TSH) >4.67 µIU/mL. Biochemical hypothyroidism (T4 < 4.5 µg/dL) did not differ significantly. Patients treated with IMRT were younger than those treated with 3DCRT (median 50 years vs. 56 years; *p* = 0.08) and received a higher dose per fraction. In multivariate analysis, age was associated with hypothyroidism (*p* = 0.02). Other factors that had a significant impact on the development of hypothyroidism were node positivity (*p* = 0.02), hypopharyngeal and laryngeal tumours (*p* = 0.01) and D100 (*p* = 0.022). D100 is the mean dose received by 100% of the contoured thyroid. A mean dose of >40.27 Gy resulted in significantly more hypothyroidism. The authors concluded that the thyroid should be seen as an organ at risk, and should therefore be delineated so the dose can be kept to a minimum, or at least be kept below 40 Gy. They also suggested that prospective randomised controlled trials should be set up to evaluate the dose-effect relationship more closely for IMRT-induced hypothyroidism. Lastly, they underlined the importance of screening patients for hypothyroidism after radiotherapy and prescribing thyroid hormone replacement when clinical hypothyroidism presents itself or when TSH levels remain elevated at successive follow-up visits.

### 4.7. Voice

The theory is that a higher dose to the vocal cords causes more oedema of the mucosa, which results in vocal cord dysfunction in the acute setting, and that this can also result in fibrosis and atrophy at the submucosal and muscular level which causes long-term speech and voice problems [20].

There is no data from randomised controlled trials to investigate the difference between conventional RT and IMRT and its effect on voice and speech of patients after RT. Kraaijenga et al. [21] did assess voice and speech after 10 years as a follow-up but did not randomise between conventional radiotherapy and IMRT. Rather, they used patients from another randomised controlled trial where two different chemotherapy regimens were compared, that were given concomitantly with RT. Of these patients, 22 were alive and disease-free and willing to take part 10 years after treatment. Of these 10 (45%) were treated with IMRT and 12 (55%) with conventional radiotherapy. 82% of patients had been treated for an oropharyngeal cancer. Perceptual evaluation and rating was performed by two speech language pathologists (SLP) who rated fragments read by the 22 patients. In addition, automatic assessment of voice quality was also performed by using a computer system. Lastly, patient-reported outcomes (PROMs) were also taken into consideration. Using questionnaires, patients were asked to score their voice and speech impairment. Although this is a subjective score, it may be the most important one, as it tells us how patients perceive their symptoms, and these will have a larger impact on quality of life than what experts or a computer system tell us. When rated by the two SLPs, 82% of patients (18 of 22) deviated from normal. Patients treated with IMRT showed significantly better scores than those treated with conventional radiotherapy (median perceptual speech intelligibility score 873 vs. 616; *p* = 0.006). Regarding automatic evaluation, no significant differences were noted between IMRT and conventional radiotherapy. Concerning PROMs, moderate but clinically relevant disabilities were found using the Voice Handicap Index (VHI) and Speech Handicap Index (SHI); 68% and 77% of patients respectively. Patients treated with IMRT showed significantly better scores over all domains than those treated with conventional radiotherapy (*p* = 0.021 for VHI and SHI). Although one could suggest that automatic assessment of voice quality is the most scientifically correct, as it is less prone to bias than the opinion of two experts, the most important aspect of this study was the scores patients gave. Therefore, we conclude that in this study, there was a significant difference in speech and voice outcomes between IMRT and conventional radiotherapy, in favour of the former. However, we have to keep in mind that this was not a randomised controlled trial and is therefore more prone to bias.

### 4.8. Dermatitis and Fibrosis

Acute dermatitis and late fibrosis are a dose-dependent phenomenon. Radio-dermatitis grade 3 or more at the end of RT, is associated with fibrosis score 2 or more when using RTOG criteria at 6 months [22]. Acute dermatitis ranges from mild erythema to moist desquamation and ulceration. Patients may complain from pain during this acute phase. Late side-effects consist of fibrosis and telangiectasia’s that present weeks to years after RT [23]. In this phase, they may experience discomfort when the skin becomes thinner and harder, and they may experience less range of motion. It is believed that IMRT may cause more-acute dermatitis because more beams are used than with 2-3DCRT, resulting in a larger volume of irradiated skin to receive an intermediate dose. The study of Gupta et al. [8] found no difference in acute dermatitis between IMRT and 3DCRT. They also investigated long-term toxicity and using the Radiation Therapy Oncology Group (RTOG) late morbidity criteria they assessed patients at 6-month intervals up to 36 months after treatment. Surprisingly, although acute dermatitis did not differ significantly between the two treatment arms, late subcutaneous fibrosis was significantly less frequent in patients treated with IMRT compared to patients in the 3DCRT-arm at all time points. Gosh-Laskar et al. [9] from the same research centre as Gupta et al., published their results in 2016. Their secondary endpoint was also late sequelae, of which subcutaneous fibrosis is one. They did not find a significant difference in late fibrosis between 3DCRT and IMRT. This finding is also supported by the PARSPORT trial, [7] where no significant difference was seen in acute dermatitis in patients treated with IMRT compared to conventional radiotherapy. A reason for these different results between studies may be other reasons for development of fibrosis, namely upfront neck dissection and N-stage [22]. Another reason may be the implementation of skin sparing techniques, which are used by centres to prevent a larger volume of skin to receive an intermediate dose with IMRT. We believe that acute dermatitis is an important toxicity because it causes a lot of pain to head and neck cancer patients. Fibrosis of the skin and neck muscles is also an important toxicity from which many patients suffer because it causes neck pain and results in a smaller range of movement of the neck. In summary, care should be taken to avoid acute dermatitis as this is a risk factor for the development of fibrosis.

### 4.9. Quality of Life

Possibly the most important outcome after loco-regional control and overall survival, is quality of life (QoL). It can be seen as a summation of all the toxicities a patient can experience after radio(chemo)therapy. It is especially important because it is a patient-reported outcome which may tell us more than a physician’s interpretation of symptoms. The number of studies that investigate QoL reflects its importance. There are several questionnaires that are validated and used frequently to score QoL.

The Medical Outcomes Short Form 36 (SF-36) is divided into eight subscales: physical and social functioning, role limitation-physical and -emotional, mental health, vitality, pain and general health perception. A higher score on a scale of 100 indicates a better health perception. The European Organization for Research and Treatment of Cancer (EORTC) core questionnaire (QLQC30) uses five functional scales (physical, emotional, social, role and cognitive), three symptom scales (pain, fatigue and nausea or vomiting) and a global QoL scale. The final score is also on 100. The EORTC head-and-neck questionnaire (QLQ-H&N35) was designed specifically for patients with HNC. There are seven scales assessing pain, social eating, social contact, speech, swallowing, senses (taste/smell) and sexuality. In addition, there are 11 single items about teeth, mouth opening, sticky saliva, dry mouth and coughing. Here, the maximum score is also 100.

Pow et al. [12] were the first to perform a randomised controlled trial to compare QoL in patients treated with 2DRT vs. IMRT in the treatment of early nasopharyngeal cancer. Fifty-one patients with T2, N0/N1, M0 tumours were included in the study. SF-36, QLQC30 and QLQ-H&N35 outcomes were used. With SF-36 they found that physical role and bodily pain were significantly better 12 months after IMRT than after 2DCRT (86.5 vs. 58.3 and 89.8 vs. 75.6 respectively; *p* < 0.05). They found no correlation between salivary flow rates and subscale scores. Using data from EORTC QLQC30, only functional role (revised) was significantly better 12 months after IMRT than after conventional radiotherapy (100.0 vs. 95.2; *p* < 0.05). A significant negative correlation was found between global health status and stimulated whole salivary flow rate, which was unexpected. No clear explanation is given for this other than that it was the result of statistical chance. However, significant correlations were found between emotional- and role-function and stimulated salivary flow rate. Using results from QLQ-H&N35, IMRT resulted in better outcomes at 2 months for sticky saliva and more weight gain. At 2 months however, IMRT resulted in more sense-problems (taste/smell), but this disappeared again at 6 and 12 months. At 6 and 12 months, IMRT did better than 2DRT regarding speech problems, swallowing, coughing and sticky saliva. Significant negative correlations were found between speech problems, dry mouth and sticky saliva and stimulated salivary flow rate. All in all, the authors concluded that IMRT did significantly better than conventional radiotherapy in terms of QoL. Nutting et al. [7] assigned 47 patients to each treatment arm (IMRT and conventional RT) in a randomised manner. Using QLQC30 and QLQ-H&N35 they investigated QoL too. Mean changes in global health status did not differ significantly between the two groups at 12 (*p* = 0.78) and 24 months (*p* = 0.14). Nor did any other subscales of QLQC30. With QLQ-H&N35 no statistically significant differences were found between conventional RT and IMRT either, although at 12 and 24 months IMRT did better than conventional RT in regard to xerostomia scores. Mean increases from baseline were 48.0 vs. 56.5 at 12 months and 34.8 and 59.3 at 24 months, respectively. A possible reason for non-significant differences may be the small number of patients. Although there were no statistically significant differences between the two treatment arms, the authors conclude that there is a clinically significant reduction of xerostomia and improved QoL with IMRT compared to conventional radiotherapy because a difference in QoL score of ten points or more is considered clinically significant. Rathod et al. [24] performed a prospective randomised trial to compare QoL in patients treated with 3DCRT compared to IMRT. 60 patients were treated of which 22 completed the questionnaires at all time points. QoL was scored using QLQC30 and QLQ-H&N35. They concluded that QoL deteriorates after radio(chemo)therapy but that after IMRT this deterioration is less and recovery is more rapid and complete than after 3DCRT. This was especially true for physical functioning (*p* = 0.02), head-and-neck pain (*p* = 0.01), coughing (*p* = 0.05), swallowing (*p* = 0.04) and mouth opening (*p* = 0.02).

## 5. Further Improvement in Radiotherapy Techniques

The evidence above underlines the advantages of more accurate radiation techniques, proving that it is safe and has the advantage of causing less toxicity compared to conventional radiotherapy techniques. We have to keep in mind however that these above-mentioned trials were conducted in high volume centres and that these centres may therefore have better oncological outcomes thanks to better quality of care, better treatment plans and treatment delivery. With more complex treatment techniques such as IMRT, great care should be taken to delineate correctly and to deliver the treatment accurately. Shift in tumour volume or change in patient anatomy could potentially form a problem due to dosimetric changes and cause treatment failure or increased toxicity. Adaptive radiotherapy (ART) may form a solution. Using sequential CT scans during radiotherapy, RT plans are adapted to adjust to anatomical changes such as weight loss, and to tumour volume changes due to tumour shrinkage. However, it is unlikely that all patients treated with IMRT need ART. ART is resource intensive and time-consuming and should only be performed in patients where it will have a clinical benefit. Brown et al. [25] developed ART risk profiles for nasopharyngeal and oropharyngeal cancers that could be used as a guide for clinical decision-making. This would allow patients to be identified who would benefit from ART, before therapy has started.

Having showed that IMRT is safe regarding oncological outcome, and has a better toxicity profile than conventional radiotherapy, the fundamentals have been laid for an even more conformal technique; intensity modulated proton therapy (IMPT). The benefit of IMPT is that most of the energy is transmitted in the last millimetres, called the Bragg peak. This results in less normal tissue irradiation on the distal side of the tumour as well as the proximal side. With the gaining popularity of IMPT and the opening of more proton centres worldwide, clinical trials with the aim of providing proof of the benefits of IMPT are also increasing. The use of ART will presumably become even more important in combination with IMPT than IMRT because anatomical and tumour changes will have a larger impact on dosimetrical distribution.

## 6. Conclusions

The most important outcome after radio(chemo)therapy in the curative treatment of head and neck cancer is overall survival and loco-regional control. Theoretically there may be an increased risk of loco-regional recurrence with more precise techniques because there is less room for error. However, Nutting [7], Ghosh-Laskar [9] and Peng [10] proved that IMRT is safe with no increased risk of loco-regional recurrence. The aim of this review was to search the literature for randomised controlled trials that specifically compared toxicity profiles between IMRT and conventional radiotherapy (3DCRT or 2DRT). We can conclude that IMRT has a superior toxicity profile compared to conventional RT for xerostomia and weight loss. Non-randomised controlled trials showed that IMRT was beneficial for voice quality and mucositis but there are no RCTs available to support these results. Fatigue was more prevalent in the IMRT arm compared to the conventional RT in the study of Nutting et al. [7], probably because the posterior fossa received a higher dose. Regarding dysphagia, no benefit was shown for IMRT compared with conventional RT in randomised controlled trials, possibly because most trials in the past focused on xerostomia and sparing of the parotid glands. We await the results of the ISRCTN25458988 trial that optimises IMRT for sparing of swallowing structures. IMRT does not seem better than 3DCRT at reducing post-radiotherapy hypothyroidism. It may even increase the prevalence of subclinical hypothyroidism compared with 3DCRT. The thyroid should be delineated and seen as an organ at risk that should be avoided if possible. On the other hand, hypothyroidism can be cured with medication, while other side effects like xerostomia and dysphagia cannot. The effect of the treatment technique on acute dermatitis and fibrosis is not clear. Lastly, studies investigating QoL show at least as good results with IMRT, compared with conventional techniques, and for some aspects a better result with IMRT.

Although for some side-effects, the benefit of IMRT on conventional RT cannot be easily proven, the majority of studies show a reduction in toxicity when using IMRT in head and neck cancer. With the Hippocratic Oath ‘first do no harm’ in mind, IMRT should be used in all head and neck cancer patients, in order to try to reduce the devastating side effects. Continuous prospective data collection on toxicity and outcome will provide us more data in the future, supporting this technological progress.
